# A Case of Spontaneous Autoamputation of Ovary in a 46-Year-Old Woman: An Uncommon Presentation (Painless Ovarian Torsion) with Unique Diagnostic and Therapeutic Challenges

**DOI:** 10.1155/2023/2165226

**Published:** 2023-12-12

**Authors:** Shahla Chaichian, Mohanna Khandan, Samaneh Rokhgireh, Sahar Hosseini, Roya Derakhshan

**Affiliations:** ^1^Endometriosis Research Center, Iran University of Medical Sciences (IUMS), Tehran, Iran; ^2^Pars Advanced and Minimally Invasive Medical Manners Research Center, Pars Hospital, Iran University of Medical Sciences, Tehran, Iran; ^3^Student Research Committee, Mazandaran University of Medical Sciences, Sari, Iran; ^4^USERN Office, Mazandaran University of Medical Sciences, Sari, Iran; ^5^Smart University of Medical Sciences, Tehran, Iran

## Abstract

This article presents a case of spontaneous autoamputation of ovary in a 46-year-old nulligravid woman with a history of rheumatoid arthritis and hypertension, who presented with secondary amenorrhea and white vaginal discharge. Despite an initial diagnosis of dermoid cyst based on ultrasound findings, subsequent laparoscopic surgery revealed a necrotized oval-shaped mass in the cul-de-sac, which was identified as the right ovary that had undergone torsion and autoamputation. This case highlights the diagnostic and therapeutic challenges associated with this uncommon presentation, which may be easily misdiagnosed. Clinicians should consider spontaneous autoamputation of ovary as a potential differential diagnosis in women presenting with adnexal masses, even if there is no prior history of abdominal pain.

## 1. Introduction

Ovarian autoamputation is a rare condition characterized by the spontaneous separation of the ovary from its normal anatomical position [1, 2]. It can occur either due to torsion, inflammation, or other unknown causes and is mostly asymptomatic [3, 4]. It has been reported to be an acquired condition and not congenital as the ovary has a different embryologic origin from other pelvic organs. The exact prevalence of ovarian autoamputation is unknown, and the incidence has been reported to be as rare as 1 in 11,421 cases [1]. Autoamputation of the adnexa is a rare occurrence in pediatric cases, but it should be considered as a potential explanation for the absence of the fallopian tube or ovary in patients undergoing surgery for various reasons [5]. The clinical presentation of ovarian autoamputation can vary from asymptomatic to severe abdominal pain and may be incidentally discovered during radiological investigations or surgical procedures [3]. Patients with a history of pelvic pain, whether chronic or intermittent, may be diagnosed with torsion or adnexal autoamputation. A fetal “pelvic mass” or “ovarian cyst” can predispose the adnexa to these conditions, which may be asymptomatic and not affect fertility, especially when the contralateral adnexa are normal. Expectant management is suitable for small, asymptomatic cysts and suspected autoamputated adnexa [5–7]. Diagnosis of ovarian autoamputation can be challenging, and it often requires the use of advanced imaging techniques such as computed tomography (CT) or magnetic resonance imaging (MRI) to confirm the diagnosis [4]. Treatment options for ovarian autoamputation include conservative management with close monitoring and follow-up or surgical intervention in cases of severe symptoms or complications such as infection or torsion [3].

In this study, we present a case of ovarian autoamputation in a 46-year-old woman, which highlights the unique diagnostic and therapeutic challenges associated with this uncommon presentation. Through this case study, we aim to contribute to the limited literature available on ovarian autoamputation, its clinical presentation, diagnosis, and management.

## 2. Case Presentation

### 2.1. Patient Information and Medical History

A 46-year-old nulligravid woman presented to the hospital with a chief complaint of secondary amenorrhea that had been present for the past 7 months. The patient reported irregular menstruation and dyspareunia with a severity of 8 out of 10, but no abdominal pain. She had a past medical history of rheumatoid arthritis and hypertension and was currently on medication including methotrexate, prednisolone, adalimumab, calcium D, nortriptyline, folic acid, and losartan. The patient also reported a history of infertility for the past 3 years, despite being in her second marriage. She was under the supervision of an infertility specialist due to infertility, and the test was sent for her amenorrhea, FSH = 35, and LH = 40, and also according to the ovarian reserve test (AMH = 0.02), she was made pregnant with IVF and donated embryo. She was referred to our center for laparoscopy for ovarian dermoid cyst. The patient reported dyschezia. There was no notable allergy history.

### 2.2. Physical Examination

In physical examination, the patient's uterus was found to be of normal size, and the cervix was nulliparous. A soft and cystic mobile mass was palpated on abdominal examination, localized in the right iliac fossa.

### 2.3. Diagnosis

The first ultrasound was performed 2 months before referral and showed mild adenomyosis, an endometrial length of 2 mm, and a heterogeneous oval-shaped mass with echogenicity, internal calcification and fat, and a size of 58 *∗* 23 mm in the right adnexa, suggestive of a dermoid cyst. The initial laboratory tests were normal, and the *β*-HCG value was negative. The patient was referred for surgery after the initial visit, but returned to the hospital after 2.5 months. The second ultrasound showed a mass in the cul-de-sac.

### 2.4. Surgical Intervention

**Figure 1:**
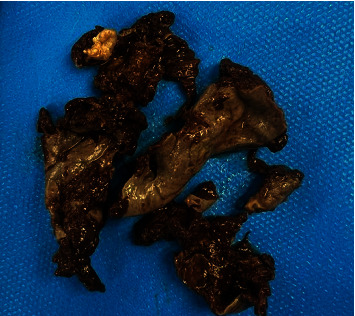
A necrotized mass of the right ovary which was removed due to torsion.

**Figure 2:**
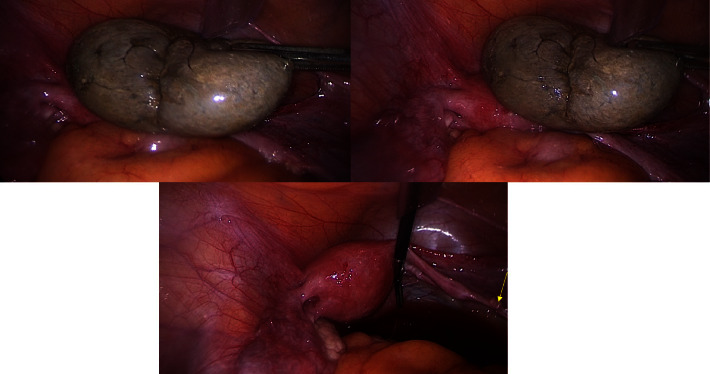
Intraoperative view of the ovary. The yellow spike indicates the presence of ovarian remnants.

The surgery was performed laparoscopically, the uterus and left fallopian tube were found to be normal. There was a 4 cm ovarian cyst which looks like a dermoid in the left side which was removed via cystectomy; the pathology of this was simple cyst. The right ovary was not seen. Half of the right fallopian tube was visible, but the rest, including the fimbriae, were not visible. In the posterior cul-de-sac, a necrotized oval-shaped mass with a size of 5 cm was found without any adhesion. The mass appeared to be the right ovary. The mass was taken out with an endobag (Figure 1). The intraoperative images are shown in Figure 2.

### 2.5. Postoperative Course

The patient's postoperative course was uneventful, and she was discharged from the hospital on the second day after surgery. She was given vaginal clotrimazole due to candida and analgesics for one week after surgery and advised to have a follow-up appointment with her surgeon in two weeks.

## 3. Follow-Up and Outcomes

Specimens received in formalin in 2 containers were labelled as follows: (a) pelvic mass consists of multiple pieces of cream rubbery tissue; (b) left ovarian cyst wall consists of multiple fragments of cream rubbery tissue. Histopathology report showed that the pelvic mass specimen was totally necrotic tissue with foci of calcification. The left ovarian cyst wall specimens were luteal cyst and simple cyst.

## 4. Discussion

Autoamputation of the ovary is a rare occurrence and is often associated with torsion of a normal ovary or an ovarian cyst, which leads to infarction and necrosis. It can be unilateral or bilateral [8]. This condition is typically discovered incidentally during ultrasound or surgery [9]. This condition can happen in adults, children, and even infants [2, 3, 10]. There have been 36 reported cases of autoamputated ovary in children ranging in age from 1 day to 12 years old, with few cases reported in adults. Pathological findings in these cases have revealed necrosis in many instances and calcification in several cases. Only a small amount of ovarian tissue was observed in the specimens of some cases. The histopathologic findings of our study also show necrotic tissue with foci of calcification [2, 11]. Various potential diagnoses were initially taken into account before the surgical decision when the patient exhibited her symptoms, including appendicitis, uterine fibroids, adnexal masses, and other gynecological and gastrointestinal issues [12].

The primary diagnosis of the patient in the first visit was dermoid cyst which eventually developed a torsion and, as a result, autoamputation of ovary. Based on the previous literature, in a retrospective study, it was shown that adnexal torsion can be caused by dermoid cyst [13]. It was shown that in most cases, 60–90 mm dermoid cysts could lead to adnexal torsion. In our study, the primary ultrasound findings show that the cyst was 53 *∗* 28 mm which is close to the previous literature. Other evidence indicates that ovarian tumors larger than 5 cm are more likely to develop torsion [14]. According to a review of the literature, an autoamputated ovary has the potential to reimplant into the omentum or peritoneum, and there is a possibility of malignant transformation. Therefore, it is suggested that all autoamputated ovaries should be excised rather than taking a wait-and-watch approach [4, 9, 11]. In a case report, an autoamputated ovarian cyst resulted in compression sequelae. Additionally, a case of autoamputated ovary with mature cystic teratoma into the cul-de-sac was described, confirmed by laparoscopy [9]. In this study, we utilized laparoscopic surgery as a therapeutic strategy for the patient [15]. It has been noted that laparoscopic surgery can also be used in the diagnosis of the autoamputation of the ovary. While ultrasonography was initially employed for its noninvasiveness and cost-effectiveness in the primary diagnosis and surgical planning, we acknowledge that laparoscopy, with its high diagnostic accuracy and therapeutic potential, is an essential tool in the evaluation of autoamputation of the ovary [6].

The patient in our study did not show significant clinical presentation of adnexal torsion. She had no signs of abdominal pain or even tenderness in the abdomen. This finding shows that ovarian autoamputation can be found incidentally without any pain. It is consistent with the previous studies showing an asymptomatic patient who was diagnosed with autoamputation of ovary [3]. The patient in our study was using corticosteroids because of the rheumatoid arthritis history. Corticosteroids can act as potent analgesic medications. This might be the reason for the relatively asymptomatic presentation of the patient in our report. So it is recommended to take a full medical history, specifically drug history, before any medical treatment considerations for patients with long-term amenorrhea and infertility. Chronic torsion can lead to the ovary being displaced from its normal position. While some patients may not show any symptoms, many of them may have episodes of acute or persistent abdominal and pelvic pain. Therefore, persistent discomfort or pain, even if it is mild, should not be ignored, especially in younger patients with cysts in both ovaries [16]. To ensure timely surgical decisions for ovaries containing cysts, including patients with chronic pain, even of low intensity, and those undergoing analgesic therapies, a vigilant approach must be maintained.

## 5. Conclusion

This case report underscores the rarity and diagnostic challenges associated with spontaneous autoamputation of the ovary, primarily caused by torsion. Autoamputation is an infrequent occurrence in women of reproductive age, with its predominant cause being torsion. Emphasis should be placed on the evaluation of patients presenting with chronic abdominal pain, and consideration should be given to the possibility that patients under chronic pain management, who may harbor ovarian cysts, warrant a proactive approach to surgical intervention to prevent symptomatic ovarian torsion, similar to the case presented herein.
